# A Change in Students' Perceptions of Peer and Faculty Attitudes to Rural Medicine following the Introduction of a Rural Health Rotation

**DOI:** 10.1155/2014/124708

**Published:** 2014-08-27

**Authors:** Martyn Williamson

**Affiliations:** Department of General Practice and Rural Health, Dunedin School of Medicine, University of Otago, P.O. Box 913, Dunedin 9054, New Zealand

## Abstract

*Introduction*. In 2000, the Dunedin School of Medicine (one of Otago Medical School's 3 clinical schools) introduced 7-week rural placement. A survey of students conducted before attending the placement showed that most students did not perceive faculty to have a positive view of rural health. In 2007, we explored whether students' perceptions had changed.* Method*. All 5th year medical students at Otago Medical School were surveyed using items from the original study. The perceptions of students in Dunedin were compared with those of students in the other clinical schools (no rural rotation) and with those of students in the original study.* Results*. In 2007, there was a significantly increased likelihood of students from Dunedin reporting perceptions of positive faculty attitudes towards rural health compared with students from the other two clinical schools and with Dunedin students from the original survey.* Conclusion*. The results suggest that student perceptions of faculty attitudes in the school towards rural health may be changed following the introduction of a general practice rural placement to its curriculum.

## 1. Introduction

Over the past two decades many countries including New Zealand have faced a shortage of medical practitioners willing to practice in rural areas [1–5]. This problem is compounded by general medical workforce shortages related to increasing demands from the combination of an ageing population and an ageing medical workforce [5–7].

Medical schools have some obligation to ensure that they produce graduates suited and attracted to areas of health need [8–10]. The two main strategies adopted by governments and medical schools worldwide to increase the supply of rural doctors have been preferential admission of students of rural origin and the use of rural undergraduate placements as part of the curriculum [11–13].

The success of these medical school based strategies will in part depend on the academic status of rural health within the schools. If rural health is seen as an acceptable discipline, it will avoid being considered as a second tier option educationally and academically and as a career choice [14, 15]. If students perceive rural health as being of low status, this will negatively influence their choice of a rural clinical school for their education [16].

**Figure 1:**
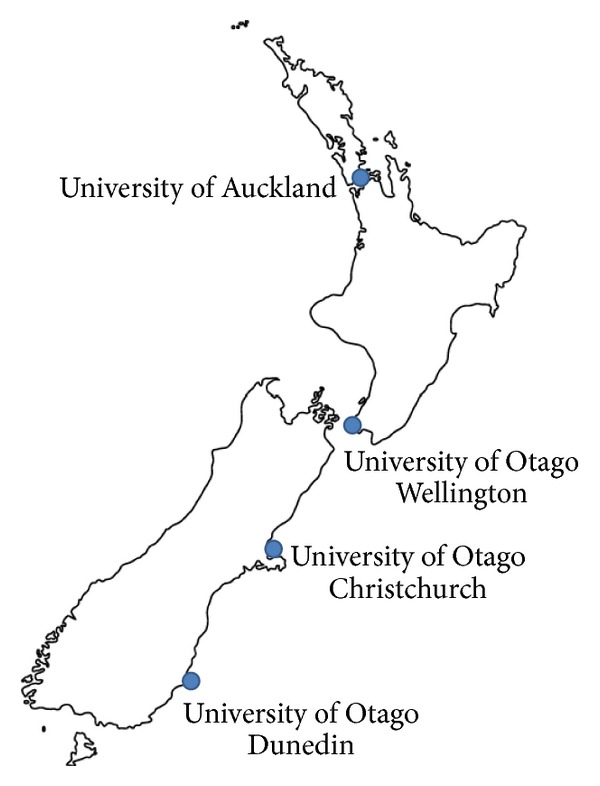


New Zealand has two medical schools, Otago and Auckland (Figure 1), which offer undergraduate courses. All Otago preclinical training takes place at the Dunedin campus. This includes a health sciences first-year course which provides students for the differing health professional disciplines. Students selected for medicine complete a further two preclinical years at Dunedin before choosing one of three Otago clinical schools (Figure 1) for their final 3 years of the 6-year course. The three Otago clinical schools have common educational objectives but autonomy over their curriculum in how these objectives are achieved.

The University of Otago, Dunedin School of Medicine, responded to local rural health workforce need [17] by introducing a seven-week rotation in rural health for all 5th year undergraduate students in 2000 [18].

A survey of the first two cohorts of undergraduates going through this rotation in 2000 and 2001 showed a positive effect of the placement on attitudes to rural health of both rural and urban background students [18]. However, at that time, the students generally felt that most of their teachers and peers did not view rural health highly as either a discipline or a career.

The aim of this study was to test whether students' perceptions of faculty and peer attitudes towards rural health at the Dunedin school in 2007 had changed compared to those of the original cohort. Our hypothesis was that student* pre-course* perceptions of faculty and peer attitudes towards rural health might be more favourable in 2007 than earlier, as a result of the increased focus on rural health within the school following the introduction of the original course. We also hypothesised that this change would not be apparent at the University of Otago's two other clinical schools based at Christchurch and Wellington which had not introduced rural undergraduate courses by the time of the study.

## 2. Method

The study was a comparison of data from a census of the 2007 cohort of 5th year medical students at each of the three clinical schools of the Otago University Medical School and data from an earlier census of the cohort of Dunedin 5th year medical students from 2000 and 2001. It was a census in that all students in each cohort were approached rather than a sample from each cohort.

We administered a hard copy questionnaire identical to that used in the 2000 and 2001 study [18]. Students were informed about the study and offered the study information, consent, and questionnaire at face to face meetings at each school. Responses were identifiable by school, allowing a comparison of the Dunedin group with their peers from the 2000/2001 survey. Students in Dunedin were surveyed in 2007* prior* to attending the 5th year rural course.

The analysis was based on a comparison of the 2007 Dunedin students' responses with responses from two other groups of students. We looked for differences between the 2007 cohort prior to the rural course and the 2000/2001 cohort prior to the rural course. We also compared the 2007 Dunedin students with their peers at the Christchurch and Wellington clinical schools, neither of which had a rural undergraduate course.

Analysis of cross-tabulation tables was based on modifications of the chi-square coefficient statistic. For nominal data (i.e., where respondents answered yes/no or rural/urban, etc.), Cramer's *V* measure was used. For ordinal data (where answers are given on a scale), Kendall's Tau-*c* measure was used.

Some questions required respondents to indicate their preference on a five-point Likert scale (e.g., not at all, not much, neutral, highly, and very highly). Due to a small number of responses in either extreme, the five-point scale was converted to a three-point scale (e.g., not at all and not much combine to become not; highly and very highly combine to become highly; in the 3-point scale, there were not, neutral, and highly).

Ethics approval for the study was provided by the University of Otago Ethics Committee low risk research involving human subjects category A (04/184).

## 3. Results

The overall response rate was 80% (193/240) but varied between the schools with Christchurch 66% (53/80), Dunedin 81% (65/80), and Wellington 93% (75/80). The response rate for the equivalent 2000/2001 survey was 88%.

When asked about their background, 74% of all respondents were identified as being of urban origin and 26% as being of rural origin. This proportion was consistent across all schools, with 81% urban respondents from Christchurch in 2007, 72% urban respondents from Dunedin (2000/2001) and 70% urban respondents in 2007, and 71% urban respondents from Wellington in 2007. We did not supply a definition of urban and rural: students self-identified into urban and rural groups (in 2000/2001, 24% of students were self-identified as rural).

We analysed by Dunedin, Christchurch, and Wellington school both students' perceptions of their teachers' (faculty) attitudes to rural practice and students' perceptions of their peers' attitudes to rural practice.  Table 1 shows that when asked “to what extent did you feel that rural practice was viewed positively by your teachers,” students at Dunedin were most likely to feel that teachers had a positive view of rural general practice, followed by students at Christchurch, with students at Wellington least likely to agree (*χ* ^2^ = 35.432 and *P* < 0.001). We did not define “teachers” leaving this open to respondents' interpretation.

When asked to what extent rural practice was promoted by their teachers, Dunedin students were also more likely to indicate that rural general practice was promoted by their teachers than students from Christchurch and Wellington (*χ* ^2^ = 42.277 and *P* < 0.001).

More Dunedin and Christchurch students than Wellington students said that rural general practice was talked about as a distinct discipline by their teachers (*χ* ^2^ = 10.777 and *P* = 0.029).

There were no differences between students in the three schools in response to the questions “was rural practice discussed as a career option by your peers?” and “was rural practice viewed highly by your peers?”

When responses from Dunedin students (prerural course) were compared between 2007 and 2000/2001, the 2007 group were significantly more likely than those in 2000/2001 to indicate that rural general practice was viewed positively and promoted as such by their teachers. Dunedin students were also significantly more likely in 2007 than in 2000/2001 to feel that their peers discussed rural general practice as a career option and that their peers viewed rural general practice positively (Table 2). The increase in the proportion of students reporting that rural practice is promoted positively by their teachers occurred through a drop in the “not promoted” group and a rise in the “promoted” group with neutral comments remaining around 30%. However, there was no significant difference in students' stated likelihood of entering rural practice between precourse responses in 2007 and precourse responses in 2000/2001 at Dunedin.

Students from rural areas reported significantly higher likelihood than students from urban areas of entering rural general practice with 11% of rural students saying they were likely to enter rural general practice, compared to 4% of urban students (Kendall's Tau-*c* = 0.299, *P* = 0.008).

## 4. Discussion

This study suggests that student and faculty attitudes to rural health (as perceived by medical students) have changed favourably over the years following the introduction of the rural curriculum at the Dunedin Medical School.

For student perceptions of faculty, this change is apparent in the Dunedin school, whereas for their perceptions of their peers the change is evident at all three clinical schools.

There has been a large shift in student perceptions of the attitudes of faculty at Dunedin school towards a positive view of rural practice over the period studied. This change is made up of reductions in both the neutral and negative responses in 2007 compared to 2000/2001. Importantly the negative dropped to 3% from 22%. This may be an indicator of a decrease in negative comments (regarding rural health from faculty to students), which have been shown to affect up to 17% of student career choices [19, 20].

The student perceptions of attitudes of faculty at Christchurch and Wellington schools in 2007 are similar to the student perceptions of faculty at Dunedin in 2000/2001 (Tables 1 and 2). The fact that the change in perceptions occurred at the school which introduced a rural health course leads to the assumption that this course has been responsible for the change. This assumption is not directly tested by our study. However the findings of change between both 2000/2001 and 2007 at Dunedin and between the three clinical schools suggest that the effect is due to the introduction of rural health as a legitimate and distinct part of the curriculum at the Dunedin school. This does not imply that the addition of a rural health placement to a curriculum will always have such an effect, as we have not explored the features or qualities of such a placement, which might contribute to such a change. Nevertheless the study does show that it is possible for changes in perception to occur. At the Dunedin school factors such as the regular highly positive student ratings of the rural course, the active involvement of other disciplines into the rural run by the rural faculty, and a positive contribution by rural faculty to medical school education processes are all likely to have played a part. It is possible that unrelated factors such as turnover in faculty may account for some perceived changes in attitude but unlikely that this change would be confined to a single school.

We do not know how the status of rural health compares with other specialties. Ideally “status” of various specialties in the medical curriculum should be equal. This would be the most professional approach for a school to promote actively and openly amongst faculty, rather than the hidden curriculum effect and its mixed messages [21]. Schools do not necessarily achieve this balance [22–24].

The attitudes of medical school faculty towards a particular discipline have been shown to affect the career intentions of medical students [19, 20, 25, 26] in both positive and negative ways. Thus the increase in positive perceptions of attitudes of faculty at Dunedin by 2007 is likely to have some influence on career intentions.

There were no significant differences between students' perceptions of their peers' attitudes towards rural health at the different clinical schools in 2007 which contrasts with the difference found between those students' perceptions of faculty attitudes. However there was a difference between student perceptions of peers in 2007 from 2000/2001, with the precourse figures for 2007 being similar to postcourse figures for the original cohort (Tables 1 and 2). This somewhat unexpected finding across the three schools in 2007 may in part be explained by the fact that all students at Otago medical school spend their first two preclinical years at Dunedin before they disperse to their clinical schools in Dunedin, Christchurch, and Wellington providing some homogeneity of experience of the student group at the school shown to have a change in perceptions of faculty attitudes. Alternatively there may be factors external to the school environment which resulted in the findings amongst the student group, though one would expect such factors to also influence faculty.

Many students formulate broad career intentions by entry to medical school or early in their undergraduate years. These career intentions have been shown to predict choice of practice [27, 28] which lends importance to factors which contribute to that early influence such as status or prestige [29]. The relevance is highlighted by findings that 45% of students adhere to their broad career preference indicated at* entry* to medical school [27] and that the most powerful predictors of a rural place of practice are rural origin combined with an intention to practice as a generalist on* entry* to medical school [12]. Prestige is known to be a more significant consideration for males, students of higher socioeconomic parents, and students of Asian origin [30–32]. Some medical disciplines have greater status than others both within the profession and the general population [33]. Lower status within the profession may be associated with increased difficulties in recruiting staff with possible reduction in quality as competition decreases [33]. So arguably, raising the profile, status, or prestige of rural health as a discipline may affirmatively influence career preference of medical undergraduates from an early stage either on entry or during medical school years [34, 35]. This may assume increasing importance in New Zealand given the high proportion of Asian origin medical students relative to the population [36, 37].

There is a potential value in increased recognition of rural health as a discipline, unrelated to workforce numbers. This may be reflected by an increase in respect for rural health practitioners and an understanding of their work place challenges from urban doctors and medical specialists. This should lead to improved communication and teamwork with benefits for patient care.

Achieving a balance in the curriculum between educational and workforce needs is a challenge. However it has been shown that quality of education does not necessarily decrease with a change in context from the traditional tertiary teaching hospital [38, 39]. Rural educational placement and affirmative rural origin entry policies may well change the student profile favourably for rural health, without compromising academic standards. An overall increase in medical school entry is required for general shortages to be addressed.

A strength of this study is that we are able to compare findings across schools and also with an earlier student cohort. Although all fifth year students at the Otago University's medical schools were involved, the numbers are still relatively small, so the study does not have the power to detect subtle changes. In addition there may be changes in the student population related to factors such as change in entry criteria, so an assumption of homogeneity between 2000/2001 groups and 2007 is not necessarily valid though the proportion of rural origin students is similar. The results represent cross sections in time rather than necessarily revealing an enduring truth. A limitation arises from the methodology. The ideal would be to utilize two parallel cohorts with baseline and follow-up measurements in both. However this was not possible at the time of the study.

There will always be a limit to the number and proportion of students who choose rural health as a career path. Increasing efforts to raise this proportion will inevitably have decreasing returns in terms of graduates actually in rural practice.

## 5. Conclusions

Students' perceptions of attitudes of faculty within a medical school towards rural health may be changed positively as a result of the introduction of a rural health placement to the curriculum. The effects on students precede their experience of the course itself. These changes are important and add to the effort at undergraduate level to increase rural workforce numbers. Further research is required to identify features of rural health placements leading to such changes before these findings can be generalised.

## Figures and Tables

**Table 1:** Students' perceptions of faculty and peers views of rural health by clinical school.

Students perception (before rural curriculum at DSM)		Christchurch School of Medicine (CSM)	Dunedin School of Medicine (DSM) (rural curriculum since 2000)	Wellington School of Medicine (WSM)	Differences between schools
		% (*n*)	% (*n*)	% (*n*)	
Rural general practice viewed positively by teachers	Highly	41.5 (22)	70.8 (46)	21.9 (16)	CSM versus DSM versus WSM
	Neutral	43.4 (23)	26.2 (17)	53.4 (39)	*χ* ^2^ = 35
	Not highly	15.1 (8)	3.1 (2)	24.7 (18)	*P* < 0.001

Rural general practice promoted by teachers	Highly	26.4 (14)	63.1 (41)	13.7 (10)	CSM versus DSM versus WSM
	Neutral	39.6 (21)	29.2 (19)	42.5 (31)	*χ* ^2^ = 42.2
	Not highly	34.0 (18)	7.7 (5)	43.8 (32)	*P* < 0.001

Rural general practice discussed as a discipline by teachers	Yes	42.3 (22)	55.4 (36)	29.3 (22)	CSM & DSM v WSM *χ* ^2^ = 10.7 *P* = 0.02

Rural general practice discussed as a career option amongst peers	Highly	76 (40)	75.4 (49)	65.3 (49)	CSM versus DSM versus WSM
	Neutral	5.8 (3)	15.4 (10)	18.7 (14)	*P* = 0.187
	Not highly	17.3 (9)	9.2 (6)	16.0 (12)	

Rural general practice viewed positively by peers	Highly	26.4 (13)	23.1 (15)	22.7 (17)	CSM versus DSM versus WSM
	Neutral	35.8 (19)	58.5 (38)	49.3 (37)	*P* = 0.822
	Not highly	37.8 (20)	18.4 (12)	28.0 (21)	

**Table 2:** Comparison of Dunedin students (precourse) perceptions of their teachers and peers views of rural health (Mann-Whitney *U* = MWU).

Student perception at DSM prior to completing rural curriculum		2000/01	2007	Statistical analyses
		% (*n*)	% (*n*)	
Rural general practice viewed positively by teachers	Highly	35.6 (31)	70.8 (46)	
	Neutral	42.5 (37)	26.2 (17)	MWU = 1709.5
	Not highly	21.8 (19)	3.1 (2)	*P* < 0.0005

Rural general practice promoted by teachers	Highly	42.5 (37)	63.1 (41)	
	Neutral	31.0 (27)	29.2 (19)	MWU = 2095.5
	Not highly	26.4 (23)	7.7 (5)	*P* = 0.003

Rural general practice discussed as career option	Yes	52.9 (46)	75.4 (49)	
	No	29.9 (26)	9.2 (6)	*χ* ^2^ = 10.633
	Do not know	17.2 (15)	15.4 (10)	*P* = 0.005

Rural general practice viewed positively by peers	Highly	11.5 (10)	23.1 (15)	
	Neutral	48.5 (42)	58.5 (38)	MWU = 2087
	Not highly	40.2 (35)	18.5 (12)	*P* = 0.002
